# Glutamate-Based Therapeutic Strategies for Schizophrenia: Emerging Approaches Beyond Dopamine

**DOI:** 10.3390/ijms26094331

**Published:** 2025-05-02

**Authors:** Mihaela Fadgyas-Stanculete, Octavia Oana Capatina

**Affiliations:** Department of Neurosciences, Discipline of Psychiatry and Pediatric Psychiatry, “Iuliu Hațieganu” University of Medicine and Pharmacy, 400394 Cluj-Napoca, Romania; mihaela.fadgyas@umfcluj.ro

**Keywords:** glutamate, glutamatergic therapies, schizophrenia, synaptic plasticity

## Abstract

Schizophrenia is a complex neuropsychiatric disorder composed of primary cluster-positive symptoms, negative symptoms, disorganization, neurocognitive deficits, and social cognitive impairments. While traditional antipsychotics primarily target dopamine pathways, they provide limited efficacy against cognitive deficits and negative symptoms. Growing evidence implicates glutamatergic dysregulation, particularly N-methyl-D-aspartate receptor (NMDA-R) hypofunction, in the pathophysiology of schizophrenia, making glutamate modulation a promising therapeutic approach. This review explores emerging glutamate-based treatment strategies, including NMDA receptor modulators, metabotropic glutamate receptor (mGluR) agents, glutamate transporter regulators, and kynurenine pathway inhibitors. We summarize preclinical and clinical findings on NMDA co-agonists (D-serine and glycine), glycine transporter inhibitors, D-amino acid oxidase inhibitors, and mGluR-targeted therapies, highlighting their mechanisms, efficacy, and limitations. In addition, we discuss novel interventions aimed at restoring glutamate homeostasis, including neuroinflammatory modulation and synaptic plasticity enhancers. Despite promising results, many glutamate-targeting therapies have yielded inconsistent clinical outcomes, underscoring the need for biomarker-driven patient selection and optimized treatment protocols. We propose that integrating glutamate modulators with existing antipsychotic regimens may enhance therapeutic response while minimizing side effects. Future research should focus on refining glutamate-based interventions, identifying predictive biomarkers, and addressing the heterogeneity in schizophrenia pathology. With continued advancements, glutamate modulation has the potential to transform schizophrenia treatment, particularly for cognitive and negative symptoms that remain largely unaddressed by current therapies.

## 1. Introduction

Glutamate, the primary excitatory neurotransmitter in the brain, plays a critical role in synaptic plasticity, learning, and memory [1,2]. Its effects are mediated through ionotropic receptors (AMPA, NMDA, and kainate receptors) and metabotropic receptors, which modulate synaptic strength and neuronal signaling pathways. Glutamate’s ability to adopt various three-dimensional conformations enables it to selectively bind to different receptor subtypes, thereby playing a crucial role in receptor-specific signaling [3].

NMDA receptor function is essential for facilitating the synaptic modifications that underpin cognitive function. Moreover, the role of glutamate in neural communication has been implicated in various neuropsychiatric disorders, particularly schizophrenia [4,5,6,7]. For example, NMDA receptor hypofunction is postulated to play a major role in the pathophysiology of schizophrenia, linking glutamate dysfunction to the disorder’s symptoms.

In this review, we first examine the role of glutamate in synaptic plasticity and cognitive function. We then explore emerging therapeutic avenues targeting various components of the glutamatergic system, including NMDA receptor modulators, metabotropic glutamate receptor agents, glutamate transporter enhancers, and modulators of the kynurenine pathway. Additionally, we discuss interventions aimed at neuroinflammation and synaptic plasticity. Finally, we consider clinical implications, safety profiles, and future directions for integrating glutamate-based strategies with existing treatments.

## 2. Glutamate and Synaptic Plasticity

A fundamental mechanism by which glutamate facilitates learning and memory is long-term potentiation (LTP), which strengthens synaptic connections following repeated stimulation [8]. Upon release into the synaptic cleft, glutamate initially activates AMPA receptors, promoting sodium (Na^+^) influx and subsequent depolarization of the postsynaptic neuron. This depolarization event removes the magnesium (Mg^2+^) block from the NMDA receptors, allowing calcium (Ca^2+^) ions to enter the cell. The resulting calcium influx triggers a series of intracellular signaling pathways that enhance synaptic efficacy by increasing AMPA receptor density at the synapse and reinforcing neural connectivity [9]. This molecular cascade underlies memory formation and experience-dependent neuroplasticity [10,11].

Conversely, long-term depression (LTD) serves as a complementary mechanism that reduces synaptic strength, thereby facilitating synaptic pruning and enhancing cognitive flexibility. LTD is initiated when synaptic activity diminishes, leading to a reduction in calcium influx and activation of phosphatases that internalize AMPA, consequently decreasing synaptic responsiveness [12]. This process is crucial for updating memory and selectively eliminating redundant neural connections [13,14,15]. Specifically, distinct forms of LTD exist beyond this NMDA receptor-dependent mechanism. Group I metabotropic glutamate receptor (mGluR)-mediated LTD can occur via different signaling cascades, and heterosynaptic LTD at inhibitory synapses results in reduced GABA release (disinhibition) of downstream circuits, as demonstrated in the hippocampus by Chevaleyre and Castillo [16].

## 3. Glutamate and Cognitive Function

Beyond its role in synaptic plasticity, glutamate is integral to higher-order cognitive functions including decision-making, attention, and executive functioning [17]. Its modulation of cortical and subcortical circuits facilitates efficient information processing and behavioral adaptation in the brain. Dysregulation of glutamatergic signaling is associated with cognitive impairments observed in various psychiatric disorders, notably schizophrenia [18,19]. Indeed, blocking NMDA receptors with agents such as ketamine induces cognitive deficits similar to those seen in schizophrenia, underscoring the critical role of NMDA-mediated glutamatergic signaling in cognition. Conversely, enhancing glutamate transmission in prefrontal cortical circuits has been associated with improved cognitive performance in preclinical models.

## 4. Glutamate Dysregulation in Schizophrenia

Neuroimaging studies employing proton magnetic resonance spectroscopy (^1^H-MRS) have revealed elevated glutamate levels in specific brain regions, such as the anterior cingulate cortex (ACC) and hippocampus in individuals with schizophrenia [20,21]. These findings support the NMDA receptor hypofunction hypothesis, which suggests that impaired NMDA receptor activity results in cortical disinhibition and excessive glutamatergic transmission [22].

Furthermore, evidence indicates that glutamate levels may serve as biomarkers for predicting the clinical outcomes of psychosis. Higher baseline ACC glutamate concentrations have been associated with a poorer response to antipsychotic treatment and an increased likelihood of symptom persistence [21]. Similarly, longitudinal studies have shown that individuals at clinical high risk (CHR) for psychosis who transition to schizophrenia exhibit elevated glutamate levels in the striatum and hippocampus prior to symptom onset [23,24]. These findings highlight the potential of glutamate-based biomarkers for early intervention and targeted treatment [25]. Research indicates that glutamate levels vary across different brain regions and patient subgroups, thereby influencing the progression of psychosis and treatment response. While some studies support the N-methyl-D-aspartate (NMDA) receptor hypofunction hypothesis, others have revealed inconsistencies, particularly in high-risk and treatment-resistant patients.

Table 1 summarizes the key findings of the major studies, including their implications for schizophrenia research and potential clinical applications.

## 5. Neuron–Glia Interactions in Glutamate Dysregulation

Neuron–glial interactions play pivotal roles in glutamate regulation in the brain. Astrocytes, which serve as primary regulatory glial cells, modulate glutamate metabolism and transmission. This is achieved through mechanisms such as the glutamate–glutamine cycle, excitatory amino acid transporters (EAATs), and release of D-serine to activate N-methyl-D-aspartate (NMDA) receptors [40]. Alterations in these processes have been strongly associated with schizophrenia phenotypes, underscoring the importance of neuroglial interactions in the pathophysiology of schizophrenia [41].

**Figure 1:**
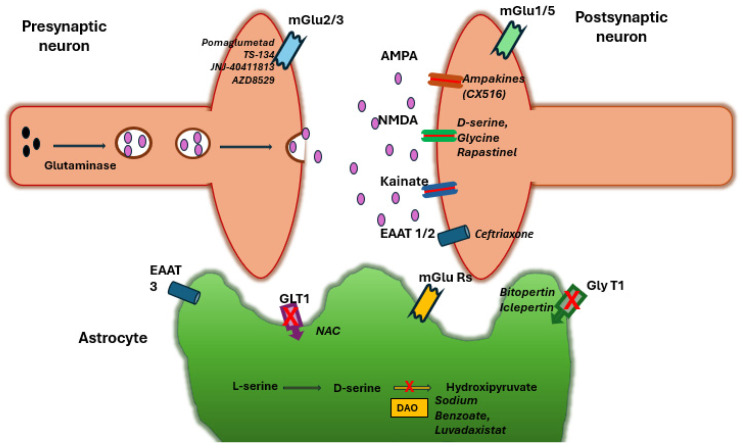
Schematic representation of the glutamatergic synapse, highlighting major receptor types and pharmacological targets. Glutamine (black spheres) and glutamate (pink spheres) are shown. Ionotropic (AMPA, NMDA, Kainate) and metabotropic receptors (mGlu1/2/3/5) are indicated, alongside key transporters (EAAT1/2/3, GLT1, GlyT1) and their modulators. Astrocytes support synaptic function through glutamate uptake (EAATs) and D-serine release. Pharmacological agents targeting glutamatergic transmission include NMDA receptor modulators (D-serine, glycine, Rapastinel, GlyT1 inhibitors, DAAO inhibitors), mGluR modulators (mGlu2/3 agonists, mGlu2 PAMs, mGlu_5_ modulators), EAAT regulators (Ceftriaxone, N-acetylcysteine), and AMPA modulators (ampakines).

A schematic overview of the glutamatergic synapse, including major receptor types and pharmacological targets, is presented in Figure 1.

To provide a comprehensive overview of various glutamate-targeting therapeutic strategies for schizophrenia, Table 2 summarizes the key treatments, their mechanisms, and clinical implications. This serves as a structured reference for understanding the different pharmacological approaches currently under investigation, highlighting their therapeutic potential and associated challenges.

## 6. NMDA Receptor Modulators

Modulation of NMDA receptors frequently through the glycine co-agonist site is a fundamental strategy in this context. The NMDA hypofunction hypothesis emerged from observations that NMDA antagonists such as phencyclidine and ketamine induce schizophrenia-like psychosis, negative symptoms, and cognitive impairment [54]. Consequently, enhancing NMDA function may mitigate these symptoms [55]. However, direct NMDA agonists pose a risk of excitotoxicity and seizures, leading most approaches to focus on indirectly augmenting NMDA receptor activity [56].

### 6.1. Glycine and D-Serine (Co-Agonists)

Glycine and D-serine interact with the glycine modulatory site (GMS) of the NMDA receptor and serve as essential co-agonists for receptor activation. Adjunctive therapy involving high doses of glycine or D-serine has demonstrated moderate improvements in overall symptoms, particularly in negative symptoms in patients [57]. For instance, meta-analyses of NMDA modulators have indicated significant reductions in residual psychotic and negative symptoms when d-serine or glycine is administered with antipsychotics [42,58]. Clinical trials have shown that d-serine (at doses of 30 mg/kg or higher) yields modest yet significant benefits for negative symptoms and cognition, although the responses vary among patients [43]. Notably, D-serine is generally well tolerated; although high doses have been associated with renal toxicity in rodents, human studies have reported only rare, reversible, and mild kidney effects [59]. No serious safety concerns were identified at the doses studied, and the combination of D-serine with a D-amino acid oxidase inhibitor (discussed below) may enhance efficacy while mitigating renal side effects. d-Cycloserine, a partial agonist of the glycine site, was also evaluated. Low-dose D-cycloserine has shown transient cognitive improvements in some studies, often in conjunction with cognitive training, but the results have been inconsistent, likely because of its complex dose-dependent effects on NMDA receptors [60,61].

### 6.2. Glycine Transporter-1 (GlyT1) Inhibitors

GlyT1 inhibitors enhance synaptic glycine levels by inhibiting their reuptake rather than by administering exogenous co-agonists. Indirect NMDA modulators have attracted considerable attention [62]. The first GlyT1 inhibitor assessed in the context of schizophrenia was bitopertin (RG1678), which has advanced to Phase III trials targeting negative symptoms [63]. Although bitopertin effectively elevated glycine levels and was well tolerated, it did not demonstrate sufficient efficacy in ameliorating negative symptoms in large-scale clinical trials [64]. More recently, iclepertin (BI 425809) has shown promising outcomes in the treatment of cognitive impairments associated with schizophrenia [44]. In a 12-week Phase II trial, adjunctive iclepertin resulted in dose-dependent enhancements in cognitive performance, as assessed by neurocognitive testing. The greatest benefit was observed at 10–25 mg, with effect sizes that distinguished it from placebo [65]. Importantly, adverse events were similar to those of placebo across all doses, indicating good tolerability [66]. However, in 6-month Phase III trials (the CONNEX program), iclepertin did not meet the primary endpoints, failing to significantly improve cognition or functioning relative to placebo [67]. Despite this setback, the drug was generally well-tolerated, with a consistent safety profile and no new safety signals.

These mixed results underscore a recurring challenge: the robust cognitive benefits observed in shorter trials can be difficult to sustain or confirm in larger studies. Other GlyT1 inhibitors (e.g., sarcosine, a natural GlyT1 substrate) have demonstrated minor beneficial effects as add-ons, and sarcosine is sometimes used off-label to treat negative symptoms [68]. Overall, GlyT1 blockade remains a promising mechanism, especially given its favorable side effect profile; however, translating pro-cognitive effects into real-world functional gains has proven challenging [57].

### 6.3. D-Amino Acid Oxidase (DAAO) Inhibitors

d-Amino acid oxidase (DAAO) is an enzyme that degrades d-serine in the brain. Inhibition of DAAO results in elevated endogenous d-serine levels, thereby indirectly enhancing NMDA receptor co-agonist activity [69]. Sodium benzoate (NaBen), a DAAO inhibitor and common food preservative, is the most extensively studied agent in this context. Clinical trials have demonstrated that adjunctive sodium benzoate treatment leads to significant improvement. In patients with chronic schizophrenia who were maintained on stable antipsychotic regimens, administration of 1–2 g/day of sodium benzoate resulted in significant enhancements in overall symptomatology, particularly in negative symptoms, when compared with placebo [70]. In a 6-week randomized trial involving 60 patients with clozapine-resistant schizophrenia, both 1 g and 2 g doses of sodium benzoate were associated with greater reductions in negative symptoms, as measured by the SANS (Scale for Assessment of Negative Symptoms) score, compared to placebo; the 2 g dose also significantly improved total PANSS (Positive and Negative Symptoms Scale) scores and QoL [71]. Patients receiving sodium benzoate exhibited improved functioning and cognitive benefits with minimal side effects. Sodium benzoate was well tolerated, with no significant adverse effects reported in these studies, making it an appealing adjunct therapy because of its safety and oral bioavailability. Luvadaxistat (TAK-831), another DAAO inhibitor, is currently under development [72]. Luvadaxistat has been shown to have pro-cognitive and pro-social effects in rodent models of schizophrenia, likely by increasing d-serine levels and enhancing NMDA receptor transmission [73]. A recent Phase II trial indicated that luvadaxistat improved electrophysiological markers of cognition, significantly enhancing mismatch negativity, an auditory event-related potential associated with NMDA function, and was predictive of cognitive outcomes in patients with schizophrenia [74].

These findings indicated that DAAO inhibition may activate the NMDA pathway in humans. Although the clinical effects on symptoms remain under investigation, preliminary indications such as trend-level cognitive improvement are promising. Similarly to GlyT1 inhibitors, DAAO blockers exhibit good tolerability. For example, in Phase I trials, luvadaxistat did not present significant side effects beyond those observed with the placebo (detailed safety results are pending publication) [73]. Overall, indirect NMDA enhancement through GlyT1 or DAAO has demonstrated potential efficacy in addressing negative symptoms and cognitive deficits; however, large-scale validation is required. These indirect methods appear to be safer than direct agonists, because they modulate NMDA activity more subtly. This conclusion is supported by the observation that indirect glycine-site modulators result in fewer adverse effects than direct agonists [75].

Previous attempts to directly modulate NMDA receptors included the use of rapastinel (GLYX-13), an NMDA receptor partial agonist that acts at a novel site and demonstrates rapid antidepressant and pro-cognitive effects in initial trials [76]. Rapastinel initially yielded positive Phase II results for cognitive deficits in schizophrenia; however, larger trials have failed to meet these endpoints. This highlights that, while NMDA modulation remains a promising area of research, not all mechanistic successes translate into clinical efficacy. Dosage, patient selection, and study design were the critical factors. New NMDA modulators, including glycine site agonists combined with transporters, DAAO inhibitors, and subunit-specific modulators, are being investigated in preclinical studies [77].

## 7. Metabotropic Glutamate Receptor Agents

Metabotropic glutamate receptors (mGluRs) are G protein-coupled receptors that play a crucial role in modulating synaptic glutamate release and postsynaptic responses. These receptors are categorized into three groups: Group I (mGlu_1_ and mGlu_5_, which are typically postsynaptic and excitatory), Group II (mGlu_2_ and mGlu_3_, primarily presynaptic autoreceptors that inhibit glutamate release), and Group III (mGlu_4/6/7/8_, which generally inhibits neurotransmitter release) [78]. Dysregulation of these modulatory receptors can lead to glutamate imbalance associated with schizophrenia. Consequently, mGluRs are promising targets for modulating the glutamatergic system. Significant advancements have been made, particularly in Group II and Group I mGluR ligands [79].

### 7.1. Group II (mGlu_2/3_) Orthosteric Agonists

Activation of presynaptic mGlu_2/3_ receptors results in a reduction in glutamate release, thereby potentially mitigating cortical hyperglutamatergia associated with NMDA receptor hypofunction [80]. Preclinical investigations have consistently demonstrated that mGlu_2/3_ agonists elicit antipsychotic-like effects in animal psychosis models. These agonists reverse behaviors induced by NMDA antagonists, such as locomotor hyperactivity, stereotypies, and sensorimotor gating deficits, attenuate the effects of hallucinogens such as DOI and enhance performance in cognitive tasks impaired by NMDA blockade [81]. The prototypical agonist LY404039, the active form of the prodrug pomaglumetad/LY2140023, exhibited efficacy in early clinical trials [45,82]. During Phase II testing, when administered as monotherapy, pomaglumetad improved total PANSS scores and ameliorated both positive and negative symptoms compared to the placebo. Notably, this was achieved without inducing extrapyramidal side effects, prolactin elevation, or weight gain, in contrast with standard antipsychotic drugs. Despite promising Phase II results, larger Phase III trials for broad schizophrenia populations were disappointing, leading researchers to halt development around 2012 [46]. However, post hoc analysis revealed that pomaglumetad benefited certain subgroups: patients in the early stages of the illness (≤3 years duration) and those who had never been exposed to atypical antipsychotics showed significant improvement [47]. This suggests that mGlu_2/3_ stimulation may be more effective in specific contexts, possibly before chronic dopamine drug exposure, or in biologically defined subtypes of ADHD [6]. Encouraged by these findings, researchers have continued to develop orthosteric agonists. TS-134 (MGS0274), a novel mGlu_2/3_ prodrug from Taisho, was tested in a ketamine challenge study. A low dose (20 mg) of TS-134 not only reduced ketamine-induced positive symptoms on the Brief Psychiatric Rating Scale (BPRS) but also normalized ketamine-induced fMRI BOLD signal changes in brain regions such as the anterior cingulate and striatum [83,84]. These pharmaco-imaging results confirm the target engagement and suggest that mGlu_2/3_ agonists can modulate glutamatergic circuits in humans at appropriate doses. Further Phase II trials on TS-134 in schizophrenia are required. Overall, the mGlu_2/3_ agonist approach remains scientifically valid, although future trials may be needed to enrich patients who are most likely to respond (e.g., early phase or certain genetic profiles).

### 7.2. Group II Positive Allosteric Modulators (PAMs)

Positive allosteric modulators (PAMs) enhance the receptor response to glutamate by binding to an alternative site rather than by directly stimulating the receptor as orthosteric agonists do. mGlu_2_ PAMs can achieve similar effects, such as reducing excessive glutamate release, with potentially fewer issues related to tolerance because they are active only in the presence of glutamate [85]. Several mGlu_2_ PAMs have progressed to clinical trials. JNJ-40411813 (ADX71149) is one such compound; it was administered to patients with schizophrenia exhibiting persistent negative symptoms in a Phase II study. Although the results have not been formally published, reports suggest that JNJ-40411813 is safe and well-tolerated in humans [86]. A post hoc analysis indicated potential improvements in attention and episodic memory in a subset of patients and a reduction in ketamine-induced negative symptom ratings in a volunteer study [87].

These findings are consistent with preclinical data, where mGlu_2_ PAMs, including JNJ-40411813 and others such as CBiPES, BINA, and TASP, demonstrated antipsychotic- and anxiolytic-like effects [88]. Another PAM, AZD8529, was evaluated in a Phase II study for schizophrenia but did not demonstrate efficacy on the Positive and Negative Syndrome Scale (PANSS) or negative symptom scales [89]. Notably, AZD8529 was tested at a single dose, which may have been insufficient to achieve complete mGlu_2_ engagement. No significant side effects, such as motor or extrapyramidal symptoms (EPS), underscore the relative safety of mGlu_2_ PAMs. This lack of efficacy may be attributed to suboptimal dosing rather than the failure of the mechanism. Consequently, follow-up studies employing higher- or multiple-dose regimens of AZD8529 and other PAMs are warranted. In summary, mGlu_2/3_ PAMs hold promise, particularly for addressing negative and cognitive symptoms but exemplify the challenges of translating preclinical success into clinical settings [90]. Patient heterogeneity suggests that future trials may benefit from selecting participants based on genetic markers such as GRM3 variants linked to glutamate function or symptom profiles to enhance the likelihood of identifying responders.

### 7.3. Group I (mGlu_5_) Modulators

The enhancement of mGlu_5_ activity has been shown to promote N-methyl-D-aspartate (NMDA) receptor function; indeed, the physiological activation of mGlu_5_ supports NMDA receptor currents and synaptic plasticity. Consequently, mGlu-positive allosteric modulators (PAMs) have been investigated as strategies for enhancing cognitive and social functions [91]. Preclinical studies have demonstrated that these deficits are induced by NMDA antagonists and can even restore synaptic plasticity in models of NMDA hypofunction. However, a significant challenge has arisen; many early mGlu_5_ PAMs result in excessive glutamate/N-methyl-D-aspartate (NMDA) activity, leading to seizures or neurotoxicity [92]. For instance, potent mGlu_5_ PAMs were found to dangerously potentiate NMDA currents, thereby limiting their therapeutic windows. This toxicity has led to the development of several clinical programs that involve these agents. In response, researchers developed “biased” mGlu_5_ PAMs that enhance mGlu_5_’s beneficial signaling pathways without overactivating NMDA receptors [93,94]. These biased modulators have demonstrated efficacy in vivo models without undesirable excitotoxic effects. One compound from the VU bloom series exhibited cognitive improvements in a rodent maternal immune activation model of schizophrenia, without inducing seizures, suggesting the potential success of this approach. Conversely, mGlu_5_ negative modulators (antagonists) have glutamate-lowering effects but tend to exacerbate schizophrenia symptoms. For example, the administration of an mGlu_5_ antagonist (MTEP) to animals induces social withdrawal and cognitive deficits similar to those caused by NMDA blockers [95]. Similarly, humans with genetic disruptions in mGlu_5_ exhibited sensory gating deficits. Therefore, careful activation of mGlu_5_ appears to be more promising than its inhibition. To date, no mGlu_5_ drug has advanced to Phase III trials for schizophrenia; however, the next generation of safer mGlu_5_ PAMs may offer a novel antipsychotic mechanism without dopamine blockade. Additionally, the mGlu₁ receptor, another member of Group I, has been less extensively studied clinically, although some preclinical research suggests that mGlu₁ PAMs may also enhance NMDA function. Rare mutations in GRM1 (encoding mGlu₁) have been identified in a few cases of schizophrenia, indicating their potential roles [96]. If mGlu₁ can be safely potentiated, it may help rectify certain circuit deficits, although drug development in this area is still in its early stages.

### 7.4. Group III (mGlu_4/8_) Approaches

Group III metabotropic glutamate receptors (mGluRs) are primarily involved in the inhibition of neurotransmitter release and are presynaptically located at the glutamate and gamma-aminobutyric acid (GABA) terminals. To date, these receptors have garnered limited attention in schizophrenia research. Broad-spectrum agonists, such as ACPT-1, a non-selective Group III agonist, have demonstrated antipsychotic-like effects in rodent models, notably reducing hyperactivity and head-twitch behaviors induced by N-methyl-D-aspartate (NMDA) receptor antagonists or hallucinogens [97]. Within Group III, mGlu_7_ and mGlu_8_ receptors are of particular interest; for instance, the genetic knockout of mGlu_8_ in mice results in behaviors analogous to schizophrenia, such as impaired fear learning [98]. The development of selective modulators of these receptors is currently underway. Currently, no Group III agents have progressed to clinical trials for schizophrenia; however, research is actively investigating whether enhancing specific mGlu_4/7/8_ functions could normalize neural network activity. Given the nascent stage of this research area, translational studies should be conducted over several years. Regarding side effects and safety, a significant advantage of mGluR-based treatments is their potential for reduced neurological side effects compared to conventional antipsychotics. Clinical trials involving mGlu_2/3_ agonists and positive allosteric modulators (PAMs) have not reported extrapyramidal symptoms, hyperprolactinemia, or metabolic syndromes. Notably, mGlu agonists, such as pomaglumetad, have not been associated with significant weight gain or motor side effects even at therapeutic doses [82]. Mild sedation or dizziness may occur, particularly with high-dose orthosteric agonists; however, the overall tolerability is favorable. Although mGlu_5_ PAMs initially exhibit dose-limiting central nervous system (CNS) toxicity, newer-biased compounds are being developed to circumvent this issue. Therefore, meticulous medicinal chemistry has been used to address the risk of seizures. Additionally, the potential for receptor desensitization due to chronic stimulation of G protein-coupled receptors (GPCRs) is a concern; however, the use of PAMs instead of direct agonists may alleviate this issue.

In summary, mGluR modulators generally exhibit a favorable side-effect profile, with the primary concern being the risk of excitatory overstimulation associated with Group I PAMs, which is currently being addressed. Genetic variations in mGluR genes such as GRM3 may influence individual responses or tolerability, suggesting that personalized approaches could optimize the safety and efficacy of these novel treatments.

## 8. Glutamate Transporters and Homeostasis

Another approach involves regulation of glutamate levels and synaptic clearance through transporters and metabolic pathways. Excessive glutamate, particularly extra-synaptic spillover, is believed to contribute to neurotoxicity and network dysfunction in schizophrenia patients [99]. Postmortem studies have identified abnormalities in astrocytic glutamate transporters in individuals with schizophrenia, such as reduced expression of the major glial glutamate transporter, EAAT2 (GLT-1), in specific regions, including the parahippocampal cortex. Insufficient glutamate reuptake results in unchecked excitatory signaling [100,101,102]. Enhancing transporter function may normalize the glutamate tone.

### 8.1. EAAT2 Upregulation (Ceftriaxone and Others)

The beta-lactam antibiotic ceftriaxone is a significant activator of EAAT2. Studies have demonstrated that ceftriaxone markedly enhances the expression and activity of GLT-1 and EAAT2 in the brain [103]. In numerous rodent models, encompassing over 100 preclinical studies, ceftriaxone treatment has been shown to reduce pathological glutamate accumulation and improve outcomes in hyperglutamatergic conditions, including ischemic stroke, amyotrophic lateral sclerosis (ALS), seizures, and addiction. Ceftriaxone has been investigated in models of schizophrenia and other related psychiatric disorders [104,105,106]. For instance, in rodents subjected to chronic stimulant exposure or NMDA antagonist administration (to simulate a schizophrenia-like hyperglutamate state), ceftriaxone normalizes glutamate levels and mitigates behavioral abnormalities, as evidenced by data from preclinical studies referenced in previous reviews [105]. The translational potential of ceftriaxone is noteworthy, given its status as an FDA-approved drug for treating infections with an established safety profile. However, challenges persist, such as the requirement for parenteral administration and uncertainty regarding the optimal dosing for central nervous system effects in humans. To date, no large-scale clinical trials of ceftriaxone in patients with schizophrenia have been conducted. A placebo-controlled clinical trial evaluating intravenous ceftriaxone for refractory psychosis enrolled only 12 participants, limiting the generalizability of its findings (ClinicalTrials. gov Identifier: NCT00591318) [107]. A 2022 review concluded that ceftriaxone may have clinical utility in acute and transient hyperglutamatergic states, such as early psychosis or substance withdrawal, where glutamate surges occur [105]. Although the safety profile of ceftriaxone is acceptable for short-term use, prolonged administration may pose risks for antibiotic-associated complications. Although EAAT2 upregulation is a promising strategy, further research is necessary to develop EAAT2 enhancers suitable for chronic use. Researchers have also explored small-molecule EAAT2 stimulators that are devoid of antibiotic activity [108]. Although none has entered clinical trials, several candidates have demonstrated the ability to enhance EAAT2 expression or function in vitro.

### 8.2. N-Acetylcysteine (NAC) and Redox Modulators

N-acetylcysteine (NAC) serves as an antioxidant precursor and plays a role in modulating glutamate neurotransmission. It supplies cysteine, which, through the cystine-glutamate antiporter (xC^−^ system), aids in the regulation of extracellular glutamate and enhances glutathione levels, thereby protecting neurons against oxidative stress [109,110,111,112]. NAC has been evaluated as an adjunctive treatment for schizophrenia that modulates the outcomes. In two double-blind, placebo-controlled trials involving individuals with chronic schizophrenia, a 12-month regimen of NAC (2 g/day) resulted in significant improvements in total symptom scores and negative symptoms compared to placebo [113,114]. Although the positive symptoms remained unchanged, improvement in the negative symptoms was noteworthy. A meta-analysis corroborated these findings, indicating modest overall benefits with the most pronounced effect observed in the reduction of negative symptoms [115]. Additionally, evidence suggests that NAC may improve cognitive function. One trial reported enhancements in working memory and attention among patients receiving NAC, whereas another study found that NAC preserved white matter integrity (fornix fractional anisotropy) in patients with early psychosis, suggesting a neuroprotective effect [116,117]. Mechanistically, NAC is believed to restore the glutamate–glutathione balance in frontal brain regions and normalize synaptic glutamate release via the xC^−^ antiporter [118]. NAC is well tolerated and available as a supplement, with side effects primarily limited to gastrointestinal disturbances or rare rashes. Given its multifaceted actions, including glutamate modulation and oxidative stress reduction, NAC is an appealing adjunctive therapy, particularly for negative symptoms associated with cortical glutamate dysregulation and oxidative damage. Ongoing research is investigating the use of NAC in early phase schizophrenia and in combination with other glutamate modulators.

### 8.3. Other Glial and Synaptic Regulators

In addition to EAAT2, other proteins involved in glutamate regulation are being investigated. EAAT1 (GLAST), another astrocytic transporter, exhibits altered regulation in schizophrenia [119]. However, specific targeting of this transporter is challenging because of the limited availability of selective compounds. Theoretically, enhancing the overall health and density of astrocytes may improve Glu uptake. For example, experimental neurotrophic treatments aim to support glial function. Another approach involves reducing the presynaptic glutamate release in hyperactive circuits, a mechanism essentially employed by mGlu_2/3_ agonists, as previously discussed. Furthermore, interventions aimed at reducing neuroinflammation may indirectly normalize glutamate levels. Activated microglia release cytokines that impair astrocytic glutamate uptake. Anti-inflammatory agents such as minocycline have demonstrated modest improvements in negative symptoms and may partially function by mitigating microglia-induced glutamate dysregulation, although direct evidence of this mechanism in patients remains limited [120]. Researchers have combined glutamate-modulating strategies to achieve additive results. One trial combined sodium benzoate (a DAAO inhibitor) with NAC in patients with persistent symptoms [51]. The rationale is that sodium benzoate increases d-serine (enhancing NMDA transmission), whereas NAC increases glutathione and may upregulate EAAT2, addressing glutamate dysfunction on multiple fronts. This combination showed some benefits in terms of symptoms and cognition (preliminary data), and importantly, both drugs were safe. Such multitarget approaches recognize that the glutamate pathology of schizophrenia is multifactorial, and concurrently addressing receptor function, synaptic release, and reuptake may be necessary for a substantial clinical impact.

Side Effects: interventions targeting glutamate homeostasis typically result in mild adverse effects. In clinical trials, ceftriaxone administration has been limited to short-term use. Apart from the common antibiotic-related effects, such as potential diarrhea or allergic reactions in susceptible individuals, no other significant adverse effects were observed. N-acetylcysteine (NAC) is considered safe as it is routinely used in gram doses for acetaminophen poisoning. Some patients have reported experiencing nausea, constipation, or dry mouth while taking NAC; however, dropout rates due to side effects remain low. Occasionally, NAC may cause a sulfurous odor in sweat or breath that may be unpleasant for some patients. As previously mentioned, sodium benzoate is well tolerated up to two grams; higher doses have not been studied, and caution is warranted for patients on sodium-restricted diets, such as those with hypertension. Generally, these metabolic modulators do not exhibit neurological side effects associated with antipsychotics, such as sedation or extrapyramidal symptoms, as they primarily act on support cells and neurotransmitter levels, rather than directly on neurons.

## 9. Kynurenine Pathway and Other Novel Strategies

### 9.1. Kynurenine Aminotransferase II (KAT II) Inhibitors

Kynurenic acid (KYNA) is synthesized by kynurenine aminotransferase (KAT) enzymes in the brain. KAT-II inhibition reduces KYNA levels and potentially alleviates NMDA receptor blockade [121]. KYN-5356, a first-in-class KAT-II inhibitor, has successfully completed Phase I clinical trials [122]. In December 2024, Kynexis Therapeutics reported that KYN-5356 was safe and well tolerated in healthy volunteers, demonstrating excellent central nervous system (CNS) penetration. Notably, the drug exhibited clear target engagement, as cerebrospinal fluid (CSF) kynurenic acid levels decreased in a dose-dependent manner, confirming KAT-II inhibition in the brain. Furthermore, the Phase I trial included exploratory assessments of cognitive function and electroencephalogram (EEG) biomarkers. KYN-5356 induced statistically significant alterations in EEG signals associated with cognition and provided preliminary evidence for enhanced cognitive performance. Although these cognitive findings should be interpreted with caution, as Phase I was not powered for efficacy, they are consistent with the hypothesis that reducing KYNA levels can enhance glutamatergic neurotransmission and brain network function. A Phase II trial involving patients with schizophrenia and cognitive impairment was scheduled to commence in 2025. If successful, KAT-II inhibition may represent a novel therapeutic approach for cognitive impairment associated with schizophrenia (CIAS), a domain inadequately addressed by current pharmacological treatments. Regarding side effects, KAT inhibitors may affect mood or other tryptophan metabolites [49]. However, the initial safety profile of KYN-5356 suggested no major psychiatric adverse effects at the tested doses. Nonetheless, careful monitoring is essential in patient trials to ensure that reducing KYNA levels, which may have neuroprotective roles in certain contexts, do not induce adverse effects, such as hyperexcitability.

### 9.2. AMPA Receptor Modulators

While NMDA receptors have traditionally been the primary focus of research, there is growing interest in α-amino-3-hydroxy-5-methyl-4-isoxazolepropionic acid (AMPA) receptors, which represent another significant class of ionotropic glutamate receptors. Cognitive processes depend on rapid AMPA-mediated transmissions [123,124]. Ampakines and AMPA-positive allosteric modulators (PAMs) have been investigated for their potential to enhance cognitive circuits [125]. Some preclinical studies have suggested that AMPA modulators counteract antipsychotic-induced cognitive slowing and improve memory encoding. For example, ampakine CX516 has been evaluated in individuals with schizophrenia and has shown some improvement in attention [126]. However, new compounds with improved pharmacokinetic profiles are currently being developed. Despite these efforts, no recent Phase II/III clinical trials in the past five years have demonstrated a significant breakthrough with AMPA modulators; thus, they remain experimental.

#### 9.2.1. Other Receptor Targets

The glutamate system is intricately connected with other neurotransmitter systems. Muscarinic M_1_/M_4_ receptors located in the interneurons can modulate glutamate release. Pharmacological agents, such as xanomeline, a muscarinic agonist, exhibit antipsychotic properties, in part, by reestablishing the acetylcholine–glutamate equilibrium. Xanomeline, now formulated with trospium in KarXT, has demonstrated a significant reduction in psychotic symptoms in Phase III clinical trials [50]. Although it is not a direct modulator of glutamate, it highlights the therapeutic potential of the pathways that indirectly affect glutamatergic activity in the brain. Furthermore, enhancement of GABAergic interneurons effectively regulates glutamate output. Certain interventions that enhance GABA function, such as positive modulators of GABA_A_ α2/3 subunits, are being investigated for their potential to ameliorate cognitive symptoms by mitigating the “noise” generated by excessive glutamate [127,128]. These strategies complement glutamate-targeting approaches by addressing the downstream effects of dysregulated Glu.

#### 9.2.2. Neuroplasticity and Others

Recent investigations into neuroplasticity have indicated that compounds that facilitate synaptic plasticity, such as those that enhance BDNF release or influence intracellular pathways, may contribute to the amelioration of glutamate network dysfunction. A pertinent example is the use of intranasal insulin or IGF-1 analogs, which have the potential to modulate NMDA receptor trafficking [52,129]. Although promising, these concepts remain in the preliminary stages of research.

Side Effects: pharmacological agents targeting the kynurenine pathway are expected to demonstrate favorable tolerability profiles based on current evidence. Given that KYN-5356 did not exhibit significant adverse effects in healthy volunteers, the primary concern was to monitor potential neuropsychiatric alterations in patients with pre-existing dysregulated glutamate levels. Specifically, an excessive reduction in KYNA levels could pose a risk of over-excitation or seizures, although such events have not yet been observed [53,130]. Common cognitive enhancers such as ampakines typically induce insomnia or headaches at elevated doses without severe adverse effects [131]. Many early ampakines were unsuccessful, primarily because of inadequate bioavailability rather than toxicity. Novel therapeutic interventions frequently encounter unforeseen effects, necessitating comprehensive phase II safety evaluation.

## 10. Conclusions

Glutamate-based strategies represent a promising therapeutic avenue for the treatment of schizophrenia, particularly for addressing symptoms inadequately managed by dopaminergic interventions, such as cognitive deficits and negative symptoms. Current methodologies, including NMDA receptor co-agonists, glycine transporter inhibitors, D-amino acid oxidase inhibitors, mGluR modulators, and glutamate transporter regulators, target distinct mechanisms within the glutamatergic system. Although several agents have demonstrated favorable safety profiles and preliminary efficacy, the clinical outcomes remain inconsistent. These disparities underscore the heterogeneity of schizophrenia and the necessity for biomarker-informed treatment strategies. Importantly, recent efforts to integrate glutamatergic modulators with existing therapies may offer synergistic benefits while minimizing adverse effects.

We propose that the advancement of glutamate-based therapeutics will depend on refining pharmacological specificity, incorporating multimodal treatment frameworks, and tailoring interventions based on the neurobiological profiles. Continued translational research and well-designed clinical trials are essential to realize the potential of these strategies for improving functional outcomes in schizophrenia.

## Figures and Tables

**Table 1:** Glutamatergic Alterations in Schizophrenia.

Study	Findings	Implications
Merritt et al. [20]	Meta-analysis found evidence of glutamatergic elevations in schizophrenia.	Supports the NMDA receptor hypofunction/disinhibition model of schizophrenia.
Bojesen et al. [25], Javitt et al. [22], Rowland et al. [26], Stone et al. [27]	Increases in cortical glutamate observed in NMDAR hypofunction produced by ketamine infusion.	Suggests that ketamine-induced glutamate increase can model aspects of schizophrenia.
Wenneberg et al. [28]	No overall difference in medial frontal Glx levels in high-risk individuals compared with controls.	Challenges previous findings of elevated glutamate in high-risk individuals.
de la Fuente-Sandoval et al. [23]	Elevated glutamate levels in the striatum in at-risk individuals who later transition to psychosis.	Early biomarker for individuals transitioning to psychosis.
Bossong et al. [24]	Elevated hippocampal glutamate levels in at-risk individuals who later transition to psychosis.	Further supports glutamate as a predictive factor for psychosis transition.
Egerton et al. [29]	Lower thalamic glutamate levels associated with continued presence of attenuated symptoms at follow-up.	Implicates thalamic glutamate levels in symptom persistence.
Demjaha et al. [30], Egerton et al. [31], Iwata et al. [32], Mouchlianitis et al. [33], Tarumi et al. [34]	Elevated glutamate or Glx in the ACC in patients with non-remission, antipsychotic resistance, or clozapine resistance.	Identifies a subgroup of patients with high glutamate levels who exibit treatment resistance.
Goldstein et al. [35]	No observed association between glutamate levels and treatment response in all studies.	Highlights inconsistencies in findings regarding glutamate and treatment response.
Egerton et al. [36]	Higher ACC glutamate levels at illness onset associated with higher likelihood of non-remission after treatment.	Glutamate levels are a predictor of treatment response in early psychosis.
Merritt et al. [37]	Non-remission associated with increases in Glx in the thalamus over 9 months.	Longitudinal changes in glutamate indicate persistent symptoms.
Jelen et al. [38]	Blunted activation of dynamic glutamate responses in ACC to cognitive task in schizophrenia and bipolar disorder.	Suggests schizophrenia involves an impaired glutamate response to cognitive demand.
Taylor et al. [39]	Blunted activation of dynamic glutamate responses in ACC to Stroop task in schizophrenia and major depressive disorder.	Identifiesblunted glutamate response as a transdiagnostic feature across psychiatric disorders.

Abbreviation: Glx = glutamate + glutamine (combined signal in ^1^H-MRS).

**Table 2:** Summary of Glutamate-Based Therapeutic Approaches in Schizophrenia.

Treatment Approach	Examples of Agents	Mechanism of Action	Potential Benefits	Challenges and Limitations
NMDA Receptor Modulators	D-serine, Glycine, Bitopertin (GlyT1 inhibitor), Rapastinel	Enhances NMDA receptor activity through co-agonists or glycine transport inhibition	Improves cognitive deficits and negative symptoms [42,43]	Variable efficacy, possible excitotoxicity risks, inconsistent trial results [44]
Metabotropic Glutamate Receptor (mGluR) Agents	Pomaglumetad, TS-134, JNJ-40411813 (mGlu2 PAM), AZD8529	Regulates glutamate transmission via metabotropic receptors	Potentially reduces psychotic symptoms and cognitive impairment [45,46]	Some agents failed in clinical trials, patient variability in response [47]
Glutamate Transporter Regulators	Ceftriaxone (EAAT2 upregulation), N-acetylcysteine (NAC)	Enhances glutamate clearance and homeostasis	Restores glutamate balance and prevents excitotoxicity	Limited human trials, difficulty translating preclinical success [48]
Kynurenine Pathway Inhibitors	KYN-5356 (KAT II inhibitor)	Reduces kynurenic acid levels to enhance NMDA function	Enhances NMDA function and cognitive processing	Potential side effects, needs further clinical validation [49]
AMPA Receptor Modulators	CX516 (Ampakine), Other AMPA-positive allosteric modulators	Enhances AMPA receptor-mediated synaptic transmission	Improves cognitive function and learning processes [50]	Limited evidence, inconsistent trial results
Neuroinflammatory modulation	Minocycline, NSAIDs, TNF-alpha inhibitors	Reduces neuroinflammation that disrupts glutamate signaling	May reduce neurotoxicity and cognitive decline	May not directly improve schizophrenia symptoms, mixed efficacy [51]
Synaptic Plasticity Enhancers	Intranasal insulin, IGF-1 analogs, BDNF enhancers	Promotes synaptic repair and neuroplasticity	Facilitates recovery by strengthening synaptic connections	Still in early research phase, needs larger clinical trials [52,53]
